# A 12-week, randomized, double-blind study to evaluate the efficacy and safety of liver function after using fermented ginseng powder (GBCK25)

**DOI:** 10.29219/fnr.v64.3517

**Published:** 2020-04-06

**Authors:** Su-Jin Jung, Ji-Hyun Hwang, Soo-Hyun Park, Eun-Kyung Choi, Ki-Chan Ha, Hyang-Im Baek, Dong-Gue Shin, Jeong-Hun Seo, Soo-Wan Chae

**Affiliations:** 1 Clinical Trial Center for Functional Foods, Chonbuk National University Hospital, Jeonju, Republic of Korea; 2 Biomedical Research Institute of Chonbuk National University Hospital, Jeonju, Republic of Korea; 3 Healthcare Claims & Management Incorporation, Jeonju, Republic of Korea; 4 Research & Development Center of GENERAL BIO Co., Ltd, Namwon, Jeollabuk-Do, Republic of Korea; 5 Department of Pharmacology, Chonbuk National University, Medical School, Jeonju, Republic of Korea

**Keywords:** fermented ginseng, liver function, gamma-glutamyl transferase, hs-CRP, anti-fatigue

## Abstract

**Background:**

Recently, clinical research has suggested that red ginseng components play a role in liver protection and combating fatigue. However, fermented ginseng has not been analyzed for liver-protective or anti-fatigue effects.

**Objective:**

This study evaluates the positive effects of fermented ginseng powder (GBCK25) on liver function.

**Methods:**

Ninety participants with elevated alanine aminotransferase levels (35 ≤ ALT ≤1 05 IU/L) were randomized to one of three groups. The participants were treated with GBCK25 tablets at a dose of 500 mg/day (high dose), 125 mg/day (low dose), or placebo group daily for 12 weeks. The primary outcomes included changes in ALT and gamma-glutamyl transferase (GGT) levels. The secondary outcomes included changes in aspartate amino-transferase (AST), high-sensitivity C-reactive protein (hs-CRP), multidimensional fatigue scale, lipid profile, and antioxidant markers.

**Results:**

In male subjects, after 12 weeks of low-dose GBCK25 (125 mg) supplementation, the GGT (*P* = 0.036) and hs-CRP (*P* = 0.021) levels decreased significantly more than those in the placebo group. High-dose GBCK25 (500 mg) supplementation significantly decreased the fatigue score compared with the placebo group. There were no clinically significant differences between the groups when studying any safety parameter.

**Conclusion:**

Our results suggest that GBCK25 supplementation has beneficial effects on liver function.

**Trial registration:**

This study was registered at Clinical Trials.gov (NCT03260543).

## Popular scientific summary

- We confirmed significant decreases in GGT and hs-CRP levels in male subjects suspected of non-alcoholic liver disease as a result of supplementation with 125 mg of GBCK25 (low dose).
- We found significant improvements in fatigue score with intake of 500 mg of GBCK25 (high dose).
- GBCK25 supplementation has beneficial effects on liver function.

Non-alcoholic fatty liver disease (NAFLD) encompasses simple steatosis, steatohepatitis, fibrosis, and cirrhosis. Its prevalence has increased with the increased prevalence of obesity and Type 2 diabetes, and NAFLD is becoming an important cause of chronic liver disease (1). Accumulation of excess fat in the liver can cause chronic inflammation, which contributes to development of chronic liver diseases such as cirrhosis and liver cancer (2). Once non-alcoholic steatohepatitis (NASH) develops, the 5- and 10-year survival rates have been estimated at 67 and 59%, respectively (3). Both NAFLD and NASH are becoming major public health issues throughout the world due to their associations with obesity, diabetes, insulin resistance, and metabolic diseases. The most common causes of liver disease and failure are Westernized dietary habits, lack of exercise, alcohol, and stress. These factors cause liver disease through oxidative stress and inflammation (4). Several prior studies have reported research and development of natural substances that may offer protection to the liver in the treatment of NAFLD (5, 6). Silymarin, a flavonoid of milk thistle (*Silybum marianum*), is widely known to exhibit liver-protective effects because of its strong antioxidant properties (7, 8). However, higher than customary doses of silymarin did not significantly reduce serum alanine aminotransferase (ALT) levels more than placebo in subjects with unsuccessfully treated chronic hepatitis C virus infection (9). Therefore, few known materials improve liver function with limited side effects. Recently, aucubin (10), ursodeoxycholic acid, and a vitamin B complex of *Plantago asiatica* have been used in the treatment of hepatotoxicity. There is ongoing global development of natural materials that offer liver protection due to lack of effective treatments for liver diseases. *Panax ginseng* C.A. Meyer is one of the most well-known medicinal plants in the world and is traditionally used in Asia to maintain physical homeostasis and enhance life energy. Many physiologically active compounds (about 40, including ginsenoside) have been identified in ginseng (11). Saponin glycoside substances, the main medicinal ingredients in ginseng, have the following biological effects: antineoplastic effects (12, 13), immunity enhancement (14, 15), blood flow improvement (16, 17), anti-fatigue and anti-stress effects (18, 19), nervous system protection (20, 21), and cognitive function improvement (22, 23). Recent clinical research has also suggested that red ginseng materials play a significant role in liver protection and combating fatigue (24). Another pre-clinical study found that administration of fermented ginseng powder (GBCK25) to an animal model of NASH led to inhibition of hepatocyte destruction and overall liver protection (25). However, there have not been sufficient randomized controlled trials to show that fermented ginseng materials have a clinically significant role in improving liver function or in treating fatigue. GBCK25 is produced by a standardized process of multiple fermentations using the strain *Saccharomyces servazzii* GB-07 and pectinase enzyme to convert general ginsenoside to compound K (26).

Therefore, the objective of this study was to evaluate the effects and safety of GBCK25 on liver function and in treatment of fatigue for those with signs of liver dysfunction.

## Material and methods

### Participants

This study was approved by the institutional review board (IRB) at our institution (approval number 2016-02-029). The entire study was conducted in accordance with the provisions of the Helsinki Declaration, as well as the standards for Korean Good Clinical Practice (KGCP). This randomized, double-blind, placebo-controlled study was conducted between July 2016 and October 2017.

The subjects in this study were recruited from the Clinic Trial Center for Functional Foods (CTCF2) at Chonbuk National University Hospital. The participants responded to advertising, including brochures, posters, and the Chonbuk National University Hospital website. Volunteers were considered eligible after providing written informed consent and undergoing a screening test (including a medical interview, physical examination, and diagnostic medical examination) within 3 weeks of the initial evaluation date (0 day). The inclusion criteria were as follows: men and women aged ≥19 and ≤70 years at the time of the screening test, serum ALT level of 35–105 IU/L, and those who had heard and fully understood the detailed description of the study and voluntarily agreed to participate. Volunteers were excluded if they met any of the following criteria:

1. Those who had taken liver function improvement medicines and/or health functional foods within the preceding 4 weeks
2. Those who were treated with antipsychotic medications within 2 months of the screening test
3. Those who took Chinese medicine within 4 weeks prior to the first dose date (However, if the medications were considered reasonable by the tester, these volunteers were allowed to participate.)
4. Those suspected of drug use or alcoholism
5. Those with a clinically significant history of hypersensitivity to drugs or health functional foods
6. Those with a past history of gastrointestinal disease (e.g. Crohn’s disease) or gastrointestinal surgery (except simple appendectomy or herniotomy) that may affect product absorption
7. Those with more than one episode of esophageal variceal bleeding, liver coma, or abdominal dropsy within 1 year of the first administration
8. Acute and chronic hepatitis (Type B and C) patients
9. Hepatitis virus (Type B and C) carriers
10. Those with signs of cirrhosis, liver cancer, or liver cancer syndrome
11. Those suffering from kidney diseases such as acute or chronic renal failure
12. Those with signs of a gallbladder pathology such as jaundice or gallstones
13. Those who were pregnant or breast-feeding
14. Fertile women who were likely to become pregnant or who did not have a reliable method of contraception (except those who had undergone infertility operations)
15. Those who participated in other human studies within 2 months of the screening test
16. Those with a serum creatinine level >2.0 mg/dL on the diagnostic examination
17. Those with any diagnostic results that the person in charge of the study found to be inappropriate for participation.

### Design, intervention, and study protocol

This randomized, double-blind, placebo-controlled intervention study was conducted at CTCF2 at Chonbuk National University Hospital. A total of 90 participants were randomized to the placebo group (1.4 g/day), GBCK25 125 mg group (low-dose group; 1.4 g/day with 125 mg/day fermented ginseng powder), or GBCK25 500 mg group (high-dose group; 1.4 g/day with 500 mg/day fermented ginseng powder). Randomization was conducted according to the allocation code of a block randomization method (randomization program of the version 9.2 SAS® system (SAS Institute, Cary, NC, USA)) at a ratio of 1:1:1 by the clinical research coordinator. Participants in both GBCK25 (test groups) and placebo groups were instructed to take the medication in the form of two tablets once a day for 12 weeks (1.4 g/day including diluent). The morning dose was taken 30 min before breakfast. All subjects were counseled to continue their typical daily activity levels and eating patterns but avoid other functional foods or dietary supplements during the 12-week study. The test products left over after the 12 weeks were used to evaluate compliance with the study protocol. To compare the drug’s effects on liver function, three tests were implemented. The first test was the screening, the second test (week 0) was conducted before the participants started taking the test products, and the third test (week 12) was conducted after completion of all 12 weeks.

The primary outcome was configured by the variation in values of ALT and GGT. The secondary outcome was determined by variations in aspartate aminotransferase (AST), total bilirubin, triglyceride (TG), total cholesterol (TC), high-density lipoprotein cholesterol (HDL-C), low-density lipoprotein cholesterol (LDL-C), inflammatory index based on high-sensitivity C-reactive protein (hs-CRP), antioxidant index based on total antioxidant capacity (TAC), and fatigue index based on the multidimensional fatigue scale (MFS) score.

### Test supplements

All samples were obtained in a refined format from GENERAL Bio Co., Ltd (Namwon, Jeonbuk, Republic of Korea). Analysis of the surface components of GBCK25 was conducted as previously described in another clinical test (25). In a pre-clinical study (25), we found that serum ALT concentration was reduced after GBCK25 supplementation in a Western diet (WD) fed mouse model, and that significant decrease in serum ALT concentration was noted in mice receiving GBCK25 administration. Based on those results, the appropriate GBCK25 dose for subjects in the present study was 125 mg/day or 500 mg/day. The clinical trial products were chosen from three types of optimal placebo (one type) and test drug (two types) taking into consideration appearance, taste, and aroma. All tablets (placebo and drug) had the same appearance of a white-coated press through pack (PTP) wrapped medicine (Table 1). Both the researchers and participants were blinded to the type of test product assigned.

**Table 1:** Composition of the test and placebo products

Ingredients	Contents (%)		
	Fermented ginseng powder 125 mg (low dose)	Fermented ginseng powder 500 mg (high dose)	Placebo
Fermented ginseng powder	8.93	35.72	–
Refined glucose	55.00	50.00	60.00
Cellulose	29.67	10.68	32.80
Ginseng flavor	0.30	–	0.50
Caramel coloring	2.50	–	3.00
Paprika color	–	–	0.10
Magnesium stearate	1.00	1.00	1.00
Silicon dioxide	1.00	1.00	1.00
Coating materials	1.60	1.60	1.60
**Total**	**100**	**100**	**100**

### Biochemical analysis

All subjects underwent validation and safety evaluations at baseline (week 0) and after completing the 12-week study. Blood was collected from all participants after at least 12 h of fasting. The blood was then centrifuged (Hanil Science Industrial Co., Ltd, Seoul, Korea) for 20 min at 3,000 rpm and stored at –70°C until the analysis. Alanine aminotransferase, gamma-glutamyl transferase (GGT), and AST were measured using an auto-analyzer (Variant; Bio-Rad, Hercules, CA, USA). The levels of TC, TG, and HDL-C in blood lipids were analyzed using an automatic blood analyzer Cobas integra 800 (Roche instrument Center AG, Rotkreuz, Switzerland). LDL-C was calculated according to the Friedewald formula (27). Total serum antioxidant capacity (TAC) was measured by using a commercial assay kit (Rel, assay diagnostics kit, Mega Tip, Gaziantep, Turkey). The kit reagent was used to measure the antioxidant index. Serum hs-CRP concentration was measured using the human hs-CRP ELISA method.

### Multi-dimensional Fatigue Scale score

Participants were evaluated for level of fatigue at baseline (week 0) and at week 12 using the MFS, developed by Schwartz et al. in 1993 (28). The MFS objectively measures the physical, psychological, and social symptoms of fatigue using 19 questions divided into the following three categories: general values (eight items), daily living disabilities (six items), and situational fatigue (five items). The total points range from 19 to133. Participants completed the MFS over the last 2 weeks and responded to each item on a seven-point scale. The total MFS score was obtained by summing the individual scores.

### Measurement of dietary intake

The dietary intake survey of the subjects was performed based on a list of food records during their first (week 0) and second (week 6) and third (week 12) visits. The participants were encouraged to record as much food as possible on the 3 days (2 weekdays and 1 weekend day) before their first (week 0), second (week 6), and third (week 12) visits. The test manager also recalled the dietary list during the first, second, and third visits. Investigation and analysis of the dietary intake data were conducted for an average of 3 days before and after clinical participation using the Can-Pro 4.0 software program (Korean Nutrition Society, Seoul, Republic of Korea).

### Safety investigation

The clinical conditions of the subjects, including adverse reactions, were evaluated and recorded in the case report list. Participants’ vital signs and their general blood and chemistry levels were also measured. Hematological parameters assessed included white blood cell (WBC), red blood cell (RBC) hemoglobin, hematocrit, and platelet count. Blood biochemical tests were conducted to assess total bilirubin, total protein, albumin, blood urea nitrogen (BUN) creatinine, glucose, creatinine kinase, and lactate dehydrogenase (LDH). Urine samples were examined for specific gravity, pH, WBC, nitrite, protein, ketone, bilirubin, urobilinogen, and occult blood. All biochemical analyses were conducted by the clinical pathology department of our hospital.

### Statistical analysis

All statistical processing was analyzed using 9.2 SAS^®^ system (SAS Institute, Cary, NC, USA). All data were presented as mean ± SD for continuous variables and as frequency for categorical variables. Intention-to-treat (ITT) and per protocol (PP) analyses were conducted. Categorical variables were compared using the Chi-square test (Fisher’s exact test). For average comparisons between the two groups, an independent sample *t*-test was used for independent samples, and a paired sample *t*-test was used for paired samples. For three or more groups, one-way ANOVA was conducted. A subgroup analysis was conducted according to the sex of the subjects. *P*-values <0.05 were considered statistically significant.

#### Sample size estimation

The sample size estimation for this human study assumed the following: a change in ALT after 12 weeks of GBCK25 500 mg (high dose) of 14.4 IU/L (μ1); a change in the GBCK25 125 mg (low dose) group of 11.3 IU/L (μ2); variation in the placebo group of 4.3 IU/L (μ3); and a standard deviation of 20 IU/L. Based on these assumptions, the result variables and designs were similar to those stated by Fried et al. (9).

## Results

### Participant characteristics

**Fig. 1:**
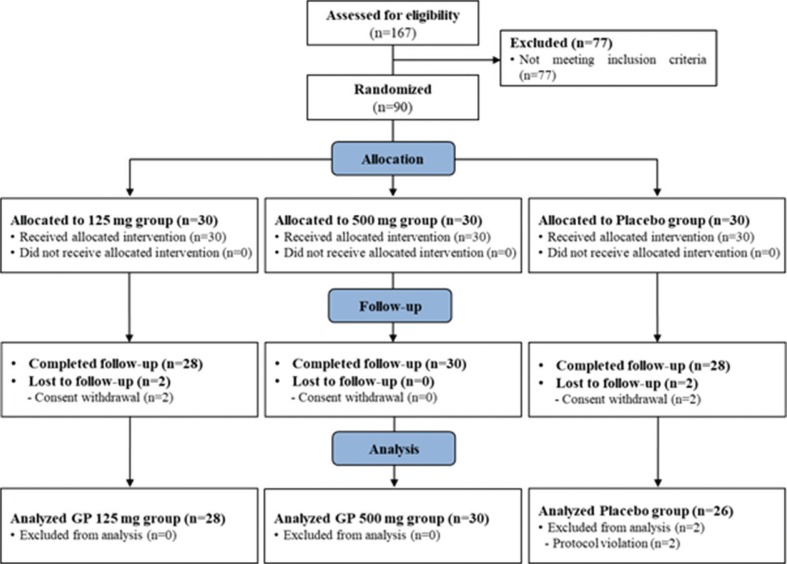
Flow diagram of the participants in this human study.

The general characteristics of the subjects in this study are presented in Table 2. There were 90 participants, of whom 90% were male (*n* = 80). The average age was 43.5 years. There were no statistically significant differences in sex, composition, age, height, BMI, liver function, or lipid profile. A total of 167 subjects were screened, and 90 subjects were ultimately enrolled. Among the 90 registered participants, four left the study prior to completion (two in the GBCK25 125 mg group and two in the placebo group). In addition, two subjects were in violation of the plan: one subject in the placebo group took prohibited drugs, and the other subject violated the exclusion criteria of the placebo group. Therefore, a total of 84 subjects (28 in the GBCK25 125 mg group, 30 in the GBCK25 500 mg group, and 26 in the placebo group) completed the specified procedures (Fig. 1).

**Table 2:** Demographic characteristics of the study participants

Variables	GBCK25 125 group (*n* = 30)^d^	GBCK25 500 group (*n* = 30)	Placebo group (*n* = 30)	Total (*n* = 90)	*P*^a^
Age, years	42.83 (11.03)	45.07 (11.38)	42.67 (10.85)	43.52 (11.02)	0.647
Sex (male/female)	26/4	25/5	29/1	80/10	0.328^b^
Drinking (yes/no)	22/8	20/10	21/9	63/27	0.853^c^
Alcohol consumption (units/week)	12.79 (10.47)	17.54 (14.59)	14.24 (14.30)	14.78 (13.13)	0.498
Current smoker (yes/no)	11/19	10/20	7/23	28/62	0.510^c^
Smoking (cigarettes/day)	15.82 (5.31)	18.50 (5.80)	16.86 (3.76)	17.04 (5.12)	0.502
Height (cm)	171.10 (7.61)	169.43 (8.34)	172.07 (6.55)	170.87 (7.53)	0.396
Weight (kg)	77.32 (11.17)	77.26 (15.50)	81.65 (12.67)	78.74 (13.25)	0.343
Body mass index (kg/m^2^)	26.38 (3.06)	26.85 (4.55)	27.48 (3.42)	26.90 (3.72)	0.520
Systolic blood pressure (mmHg)	125.07 (12.83)	121.03 (13.22)	124.53 (10.27)	123.54 (12.11)	0.341
Diastolic blood pressure (mmHg)	79.87 (10.32)	80.33 (9.89)	79.47 (7.72)	79.89 (9.31)	0.822
ALT (IU/L)	54.11 (18.07)	56.00 (19.18)	54.23 (19.00)	54.82 (18.55)	0.912
GGT (IU/L)	72.32 (56.32)	98.00 (98.05)	65.81 (52.29)	79.48 (73.58)	0.218
AST (IU/L)	35.96 (11.52)	35.10 (9.10)	35.65 (9.45)	35.57 (10.02)	0.920
Total cholesterol (mg/dL)	201.11 (42.02)	217.93 (33.85)	196.46 (28.26)	205.17 (34.71)	0.540
Triglycerides (mg/dL)	167.18 (84.95)	256.57 (220.55)	196.46 (28.26)	173.40 (111.26)	0.410
HDL-C (mg/dL)	49.32 (11.25)	49.40 (10.02)	47.15 (13.29)	48.62 (11.52)	0.864
LDL-C (mg/dL)	119.93 (37.51)	123.57 (36.35)	114.42 (27.69)	119.31 (33.85)	0.420
hs-CRP (mg/L)	2.31 (4.52)	1.96 (3.88)	1.63 (2.19)	1.96 (3.53)	0.310
TAC (mmol/L)	1.40 (0.12)	1.36 (0.20)	1.33 (0.22)	1.36 (0.18)	0.862
MFS score^e^	69.93 (16.69)	75.03 (14.61)	63.85 (16.21)	69.60 (15.84)	0.742

Values are presented as mean (SD) or number.

a Analyzed by one-way ANOVA.

b Analyzed by Fisher’s exact test.

c Analyzed by chi-square test.

d GBCK25 125 group: fermented ginseng powder 125 mg group, GBCK25 500 group: fermented ginseng powder 500 mg group.

e Range 19–133.

## Efficacy evaluation

### Blood test

**Fig. 2:**
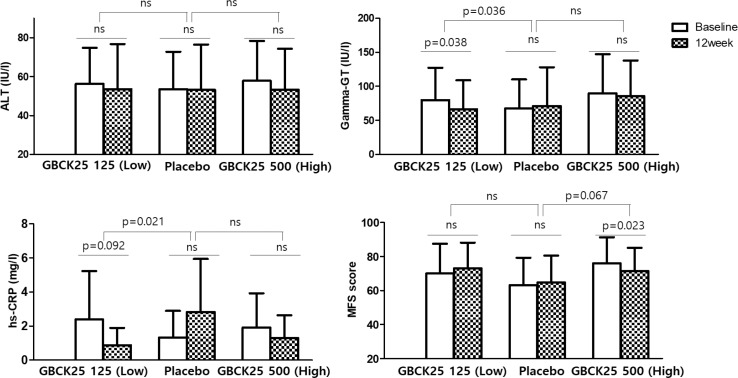
Changes in liver enzymes, hs-CRP, and MFS score of the male subjects. There was a difference in changes of GGT and hs-CRP levels between the GBCK25 125 (low dose) and placebo groups. The mean MFS score was significantly decreased in the GBCK25 500 group (high dose) after 12 weeks.

Table 3 represents changes in liver function index and lipid profile measured before and after 12 weeks of participation. After 12 weeks of intake, there was no statistically significant difference in ALT between the intake groups. There was a significant decrease in GGT in the GBCK25 125 mg (low dose) group after 12 weeks of intake (*P* = 0.049); however, the difference in decrease compared with the placebo group was not statistically significant (*P* = 0.060). Figure 2 represents the results from men after excluding those suspected of alcoholism (characterized by a GGT > three times the upper limit of normal range, *n* = 2). Women were excluded from this analysis because their liver function may be protected from non-alcoholic hepatic injury by estrogen (*n* = 10). In men, there was no statistically significant difference in ALT across groups. However, after 12 weeks of intake, the change in GGT in the GBCK25 125 mg (low dose) group significantly decreased by –13.50 ± 29.95 IU/L compared with the placebo group (which increased by 3.24 ± 24.15 IU/L) (*P* = 0.036). In the GBCK25 500 mg (high dose) group, GGT decreased by −3.91 ± 25.32 IU/L (*P* = 0.466) after 12 weeks of intake, which was not significantly different from that of the placebo group. There were no statistically significant changes in AST, TC, TG, HDL-C, and LDL-C across the intake groups at week 12.

**Table 3:** Primary and secondary outcome measures between baseline and final weeks of treatment

	GBCK25 125 group (*n* = 28)^g^	GBCK25 500 group (*n* = 30)	Placebo group (*n* = 26)	*P*^b^	*P*^c^	*P*^d^
ALT (IU/L)						
Baseline	54.11 (18.07)	56.00 (19.18)	54.23 (19.00)			
12 weeks	54.36 (22.37)	52.23 (22.33)	52.88 (22.90)			
Change	0.25 (22.56)	−3.77 (19.03)	−1.35 (14.35)	0.756	0.598	0.722
*P*^a^	0.954	0.287	0.637			
GGT (IU/L)						
Baseline	72.32 (56.32)	98.00 (98.05)	65.81 (52.29)			
12 weeks	61.25 (41.46)	94.40 (96.34)	68.54 (66.74)			
Change	−11.07 (28.42)	−3.60 (22.14)	2.73 (23.80)	0.060	0.307	0.131
*P*	0.049	0.381	0.564			
AST (IU/L)						
Baseline	35.96 (11.52)	35.10 (9.10)	35.65 (9.45)			
12 weeks	35.86 (12.96)	33.87 (11.81)	34.35 (12.60)			
Change	−0.11 (10.13)	−1.23 (11.47)	−1.31 (9.49)	0.656	0.979	0.891
*P*	0.956	0.561	0.489			
TC (mg/dL)						
Baseline	201.11 (42.02)	217.93 (33.85)	196.46 (28.26)			
12 weeks	204.82 (41.33)	216.07 (38.88)	203.62 (29.46)			
Change	3.71 (20.24)	−1.87 (35.65)	7.15 (20.47)	0.538	0.244	0.448
*P*	0.340	0.776	0.087			
TG (mg/dL)						
Baseline	167.18 (84.95)	256.57 (220.55)	196.46 (28.26)			
12 weeks	202.43 (107.60)	243.97 (190.87)	203.62 (29.46)			
Change	35.25 (95.17)	−1.87 (35.65)	7.15 (20.47)	0.538	0.244	0.448
*P*	0.060	0.776	0.087			
HDL-C (mg/dL)						
Baseline	49.32 (11.25)	49.40 (10.02)	47.15 (13.29)			
12 weeks	49.11 (11.36)	49.37 (10.01)	49.12 (13.02)			
Change	−0.21 (5.76)	−0.03 (7.46)	1.96 (6.65)	0.203	0.299	0.420
*P*	0.845	0.981	0.145			
LDL-C (mg/dL)						
Baseline	119.93 ± 37.51	123.57 ± 36.35	114.42 ± 27.69			
12 weeks	117.68 ± 36.90	122.57 ± 42.80	118.12 ± 26.67			
Change	−2.25 (21.76)	−1.00 (35.07)	3.69 (19.93)	0.301	0.535	0.695
*P*	0.589	0.877	0.354			
TAC (mmol/L)						
Baseline	1.40 (0.12)	1.36 (0.20)	1.33 (0.22)			
12 weeks	1.38 (0.14)	1.32 (0.22)	1.31 (0.18)			
Change	−0.02 (0.13)	−0.04 (0.17)	−0.02 (0.18)	0.989	0.656	0.857
*P*	0.515	0.232	0.656			
hs-CRP (mg/L)						
Baseline	2.31 (4.52)	1.96 (3.88)	1.63 (2.19)			
12 weeks	1.02 (1.21)	1.24 (1.32)	2.75 (5.03)			
Change	−1.29 (3.92)	−0.71 (3.95)	1.12 (4.96)	0.052	0.129	0.104
*P*	0.093	0.332	0.259			
MFS score						
Baseline	69.93 (16.69)	75.03 (14.61)	63.85 (16.21)			
12 weeks	74.11 (18.15)	69.60 (15.61)	65.50 (15.12)			
Change	4.18 (15.43)^e^	−5.43 (12.14)^f^	1.65 (12.95)^e^	0.520	0.039	0.024
*P*	0.164	0.021	0.521			

Values are presented as mean(SD).

a Analyzed by paired *t*-test.

b Analyzed by independent *t*-test (difference between change in the GBCK25 125 group vs. placebo group).

c Analyzed by independent *t*-test (difference between change in the GBCK25 500 group vs. placebo group).

d Analyzed by one-way ANOVA (difference between change in the GBCK25 125 vs. GBCK25 500 vs. placebo group).

e,f Values with different letters differ significantly (*p* < 0.05) among the three groups by post-hoc Bonferroni correction.

g GBCK25 125 group: fermented ginseng powder 125 mg group, GBCK25 500 group: fermented ginseng powder 500 mg group.

### hs-CRP and TAC

Table 3 represents changes in hs-CRP index and TAC after 12 weeks of intake of the test products. The hs-CRP decreased after 12 weeks of GBCK25 125 mg (low dose) treatment; however, this difference was not statistically significant compared with the placebo group (*P* = 0.052). There was no statistical significance in TAC difference between intake groups and placebo before or after 12 weeks of the test products. When studied in male participants alone, the hs-CRP decreased by –1.51 ± 4.20 mg/L in the GBCK25 125 mg (low dose) group after 12 weeks, while that of the placebo group increased by 1.51 ± 4.64 mg/L (*P* = 0.021). This subgroup analysis excluded participants suspected of having alcoholic hepatic injury (those with a blood GGT greater than 3× the upper limit of normal range, *n* = 2) and women (who may be protected from non-alcoholic hepatic injury by estrogen, *n* = 10). In the GBCK25 500 mg (high dose) group, the hs-CRP decreased by 0.62 ± 4.29 mg/L (*P* = 0.493) after 12 weeks, but there was no significant difference compared to that of the placebo group (Fig. 2). There was no statistically significant difference in TAC within the intake groups or between the intake groups before and after 12 weeks of the test products.

### MFS score

Table 3 shows the changes in MFS score after 12 weeks of test product use. After using the test products for 12 weeks, MFS score for the GBCK25 500 mg (high dose) group decreased significantly compared to that of the placebo group (*P* = 0.039). In the GBCK25 500 mg (high dose) group, the MFS score decreased significantly from baseline to after 12 weeks of treatment (*P* = 0.021). The MFS score significantly improved in the GBCK25 500 mg group compared to that of the GBCK25 125 mg and placebo groups (*P* = 0.024). When analyzed among men only (excluding women and participants suspected of having alcoholic hepatic injury, as above), the MFS score decreased in the GBCK25 500 mg (high dose) group after 12 weeks of treatment (compared to the placebo group), although the difference was not statistically significant (*P* = 0.067). In the GBCK25 500 mg (high dose) group, however, the MFS score decreased significantly between week 0 and week 12 (*P* = 0.023) (Fig. 2).

## Estimate of diet intake

Participants’ average daily intakes of total calories, carbohydrates, lipids, proteins, and dietary fibers were analyzed before and after 12 weeks of test product use. There was no statistically significant difference within the intake groups or between the intake groups for any item (data not shown).

## Safety

No adverse reactions were reported with the study drugs. There were no significant changes in the following clinical factors across the three groups before and after the study: electrocardiogram, vital signs (BP and pulse), or laboratory tests (WBC, total bilirubin, albumin, BUN, creatinine, uric acid, RBC, hematocrit, total protein, glucose, platelet, and pH). All clinical results were within the normal ranges (data not shown).

## Discussion

The liver function index GGT (*P* = 0.060) and inflammatory index hs-CRP level (*P* = 0.052) decreased in adults who took GBCK25 125 mg (low dose) per day for 12 weeks, although these values were not significantly different from those on placebo. When men were analyzed separately (excluding women and those with a blood GGT greater than three times the upper limit of normal range, *n* = 2), we found that GGT (*P* = 0.038) and hs-CRP (*P* = 0.021) levels decreased significantly in the GBCK25 125 mg (low dose) group compared to those of the placebo group. A prior clinical study showed that GBCK25 inhibited lipid accumulation in an animal model of hepatic injury (due to a high fat diet) and therefore protected against NAFLD (25). These effects may have reduced accumulation of fatty acids in the liver and slowed hepatocyte damage. GBCK25, which includes the major ginsenoside compound K, reduces AST and ALT levels in the serum, controls the Jun N-terminal kinase (JNK) signaling pathway, and inhibits expression of cytochrome peroxidase 2E1 (CYP2E1). Another animal study showed that fermented ginseng showed better liver protection than did fermented red ginseng in a hepatic injury animal model. The compound K content of fermented red ginseng was about 0.3 mg/g (29), whereas that of GBCK25 in this study was about 23.32 mg/g (25), much higher than that of fermented red ginseng.

This result may be explained by compound K, one of the major ginsenoside constituents of fermented ginseng, playing an anti-inflammatory role by inhibiting signal transmission of JNK in the liver (30). Kim et al. reported (31) that oxidative stress in the liver leads to inflammation via JNK activation, promoting inflammatory cytokine production. In general, an outbreak of NAFLD is caused by increased inflow of fatty acids from fat tissues to the liver due to insulin resistance at the periphery. The liver is thought to have an excessive inflammatory reaction in conditions of persistent oxidative stress, with neutral fat accumulating in the cytoplasm of hepatocytes (32). Oxidative stress can amplify inflammatory reactions in the liver when fat has accumulated in excess. These inflammatory reactions lead to chronic inflammation in the liver, which includes hepatocyte ballooning and cell death. Eventually, fibrosis develops with inflammatory infiltration and collagen deposition. GGT, mainly in the serum, exists in the outer cell membranes in several organs in the human body and is an enzyme that plays an important role in antioxidant action by maintaining a high concentration of glutathione (GSH) in the cell (33). A factor that causes hepatic injury increases GGT according to increase in oxidative stress (34). The concentration of serum GGT in patients with hypertension and diabetes is a risk factor for metabolic disease regardless of alcohol intake and liver disease. In addition, an increase in blood GGT can be a predictor of cardiovascular diseases (34–37). In general, GGT and ALT are the main markers in fatty liver which are related to insulin resistance, Type 2 diabetes, and metabolic syndrome (38–43). Lee and Lau (44) found that ginseng extracts and ginsenosides increase anti-inflammatory and anti-proliferative activities by significantly inhibiting inflammation caused by tumor necrosis factor-alpha.

Inflammatory response markers play a central role in NAFLD progress. C-reactive protein and hs-CRP are markers of simple fatty liver and NASH, respectively (45). In this study, the significant decreases in GGT and hs-CRP levels with GBCK25 125 mg (low dose) are believed to be related to compound K, a ginsenoside component of GBCK25 125 mg that has anti-inflammatory effects on the liver. A recent study identified a link between increasing hepatic enzymes and the inflammatory index. In this study, ALT and AST were not related to hs-CRP level, even though serum GGT correlates closely with the hs-CRP level. Prior studies have shown a positive correlation between GGT and hS-CRP in patients with metabolic syndrome (33, 42). Therefore, we suspect that compound K-containing GBCK25 may offer significant liver protection by reducing the oxidative stress marker GGT and the inflammatory index hs-CRP. Fatigue is the most common symptom in patients with liver disease and has an important effect on quality of life. Although the underlying cause of fatigue in liver diseases is not well-understood, the diseased liver itself is thought to change central nerve transmission (46). We found that subjects who took GBCK25 500 mg (high dose) had a significantly reduced MFS score compared to those in the placebo group. The results of these studies have assumed that ginseng or compound K components including saponins contained in GBCK25 protect against reducing oxidative stress and inflammation. This suggests that GBCK25 may play a positive role in liver-associated fatigue. Similarly, Kim et al. (19) found that patients with chronic fatigue had significant improvement in symptoms (based on mental NRS score) after taking *Panax ginseng* C.A. Meyer (1 g or 2 g/day) for 4 weeks. Another group found that overweight NAFLD patients who took 3 g of Korean red ginseng daily for 3 weeks had significantly decreased levels of adiponectin and proinflammatory cytokines (24). Our results suggest that GBCK25 500 mg improves fatigue in subjects with liver disease. GBCK25 is thought to improve fatigue by increasing total glutathione (GSH) content and glutathione reductase (GSH-Rd) activities based on the major antioxidant properties of the ginsenoside in the saponin system of ginseng (47).

The major ginsenoside contained in the ginseng-fermented powder, compound K, is considered the major contributor in treatment of fatigue through its antioxidant effects. Therefore, through this human study, we identified a tendency for GBCK25 125 mg (low dose) intake to improve blood GGT and hs-CRP levels in adults with liver failure. In particular, there were significant decreases in GGT and hs-CRP levels in male subjects suspected of non-alcoholic hepatic injury and significant improvements in MFS score with intake of GBCK25 500 mg (high dose). In addition, there were no clinically significant adverse reactions observed during this clinical study. One limitation of this study is that fatty liver was not diagnosed via the gold standard method of liver biopsy. There may also be a limitation in generalizing the results of the improvement assessment by simply indicating the liver enzyme index, a hematological test. Therefore, detailed and corroborative studies are needed to diagnose and assess the efficacy of more precise fatty liver diseases in the future.

Ultimately, we found that intake of GBCK25 intake for 12 weeks is safe and has the potential to improve liver function in patients with liver disease. GBCK25 intake may also have antioxidant action, reduce the risk of cardiovascular disease, and reduce fatigue.
